# Polyphenols Derived from Lychee Seed Suppress Aβ (1-42)-Induced Neuroinflammation

**DOI:** 10.3390/ijms19072109

**Published:** 2018-07-20

**Authors:** Yong Tang, Rui Xiong, An-Guo Wu, Chong-Lin Yu, Ya Zhao, Wen-Qiao Qiu, Xiu-Ling Wang, Jin-Feng Teng, Jian Liu, Hai-Xia Chen, Jian-Ming Wu, Da-Lian Qin

**Affiliations:** 1 Laboratory of Chinese Materia Medica, Department of Pharmacology, School of Pharmacy, Southwest Medical University, Luzhou 646000, China; tangy1989@yeah.net (Y.T.); rxiong2017@sina.com (R.X.); wag1114@foxmail.com (A.-G.W.); zhaoya129@126.com (Y.Z.); fanny.qiu@outlook.com (W.-Q.Q.); wangxiulinbest@gmail.com (X.-L.W.); 18883178848@163.com (J.-F.T.); belkn@icloud.com (J.L.); dna0805001@163.com (H.-X.C.); jianmingwu@swmu.edu.cn (J.-M.W.); 2 Institute of Cardiovascular Research, the Key Laboratory of Medical Electrophysiology, Ministry of Education of China, Collaborative Innovation Center for Prevention and Treatment of Cardiovascular Disease of Sichuan Province, Southwest Medical University, Luzhou 646000, China; 3 Department of Human Anatomy, School of Preclinical Medicine, Southwest Medical University, Luzhou 646000, China; 8056ycl@swmu.edu.cn

**Keywords:** Aβ, AD, lychee seed, neuroinflammation, catechin, procyanidin A2, apoptosis

## Abstract

Amyloid-β (Aβ) is commonly recognized as the most important factor that results in neuronal cell death and accelerates the progression of Alzheimer’s disease (AD). Increasing evidence suggests that microglia activated by Aβ release an amount of neurotoxic inflammatory cytokines that contribute to neuron death and aggravate AD pathology. In our previous studies, we found that lychee seed fraction (LSF), an active fraction derived from the lychee seed, could significantly improve the cognitive function of AD rats and inhibit Aβ-induced neuroinflammation in vitro, and decrease neuronal injuries in vivo and in vitro. In the current study, we aimed to isolate and identify the specific components in LSF that were responsible for the anti-neuroinflammation effect using preparative high performance liquid chromatography (pre-HPLC), liquid chromatography-mass spectrometry (LC-MS), and nuclear magnetic resonance (NMR) methods. To this end, we confirmed two polyphenols including catechin and procyanidin A2 that could improve the morphological status of BV-2 cells and suppress the release, mRNA levels, and protein expression of pro-inflammatory cytokines such as tumor necrosis factor-α (TNF-α), interleukin-1β (IL-1β), and interleukin-6 (IL-6) through downregulating the nuclear factor-κB (NF-κB) signaling pathway using ELISA, RT-PCR, and Western blotting methods. Furthermore, catechin and procyanidin A2 could inhibit Aβ-induced apoptosis in BV-2 cells by upregulating Bcl-2 and downregulating Bax protein expression. Therefore, the current study illustrated the active substances in lychee seed, and first reported that catechin and procyanidin A2 could suppress neuroinflammation in Aβ-induced BV-2 cells, which provides detailed insights into the molecular mechanism of catechin and procyanidin A2 in the neuroprotective effect, and their further validations of anti-neuroinflammation in vivo is also essential in future research.

## 1. Introduction

Alzheimer’s disease (AD) is the most common type of late-onset dementia, which is characterized by dysfunction in cognition and behavior. Senile plaques (SPs) and neurofibrillary tangles (NFTs) are recognized as two key pathological features of AD, and amyloid-β (Aβ) and hyperphosphorylated tau are the two main aggregated proteins in SPs and NFTs, respectively. These aggregated proteins accumulate in neurons, which lead to neuronal death and ultimately dementia. Therefore, AD patients exhibit an extensive loss of synapses and neurons in the cortex and hippocampus [1,2].

AD pathogenesis is not the only Aβ- and tau-induced neuronal injury, but is also a kind of neuroinflammation activated by Aβ and tau [3,4,5,6]. Microglia are a special form of macrophage, which are involved in the innate immune system of the central nervous system (CNS) and mediate the key neuroinflammatory cytokines [7,8,9]. In general, aggregated proteins such as Aβ, α-synuclein, and mHtt associated with AD, Parkinson’s disease (PD), and Huntington’s disease (HD) respectively, can activate microglia and release amounts of pro-inflammatory cytokines such as tumor necrosis factor alpha (TNF-α), interleukin-1β (IL-1β), interleukin-6 (IL-6), nitric oxide (NO), and reactive oxygen species (ROS). These toxic inflammatory cytokines then induce microglial activation and neuronal death, which in turn further aggravate the inflammatory response. In AD patients, the morphology of microglia has been found to be abnormal and over-activated by oligomeric Aβ [9,10].

With the improvements in the material standards of people’s lives, the aging population is becoming an important issue. More and more age-related diseases—such as dementia, diabetes, hypertension, coronary heart disease, etc.—are accelerating, of which, dementia with dysfunction in cognition and behavior brings a heavy spirit and economic burdens to the patients, their families, and countries. However, up until now, there are still no effective drugs that can be used for the treatment of AD owing to its complicated mechanisms [11]. Traditional Chinese medicines (TCMs) have a long usage history in the prevention and treatment of diverse diseases. There are many Chinese medical herbs and formulas—such as *Radix polygalae*, *Radix ginseng*, *Ginkgo biloba*, *Salvia miltiorrhiza*, *Ginseng*, Dangguishaoyao San, Tiaoxin Fang, and Zhiling Tang—that have been proven to modulate neurodegenerative disease [12,13,14,15,16,17,18].

Lychee seed (lizhihe in Chinese) is from the dried mature seeds of *Litchi chinensis Sonn*, which is a Chinese folk medicine recorded in “Ben cao gang mu”. Its traditional efficacies include promoting the circulation of Qi to resolve stagnation and treating hernia. Modern pharmacological studies have proven that lychee seed exerts the following bioactivities including improving blood sugar concentration, liver-protection, anti-oxidation, anti-virus, and anti-tumor effects. In our previous studies, we found that LSF, a bioactive fraction derived from lychee seed, could improve cognitive function of AD rats, suppress apoptosis in Aβ-induced PC-12 cells, and reduce Aβ and tau proteins in T2DM rats [19,20]. Recently, we have characterized the detailed components in LSF and proven that LSF could suppress Aβ-induced neuroinflammation in BV-2 cells. In the present study, we aimed to isolate and identify the components in LSF that are responsible for the anti-neuroinflammation effect in Aβ-induced BV-2 cells. Through its isolation, purification, identification, and elucidation using column chromatography, preparative high performance liquid chromatography (Pre-HPLC), and nuclear magnetic resonance (NMR) technologies, two polyphenols including catechin and procyanidin A2 were proven to suppress Aβ-induced neuroinflammation and apoptosis in Aβ-induced BV-2 cells. These findings in the current study provide evidences for the action substances and mechanism of lychee seed in the improvement of the cognition and behavior of AD rats, and further investigation of their pharmacokinetics in rat brain and the validation of their anti-inflammation effect in vivo are also essential for future therapeutic approaches.

## 2. Results

### 2.1. Isolation, Purification, Identification, and Elucidation of Catechin and Procyanidin A2 in LSF

**Figure 1:**
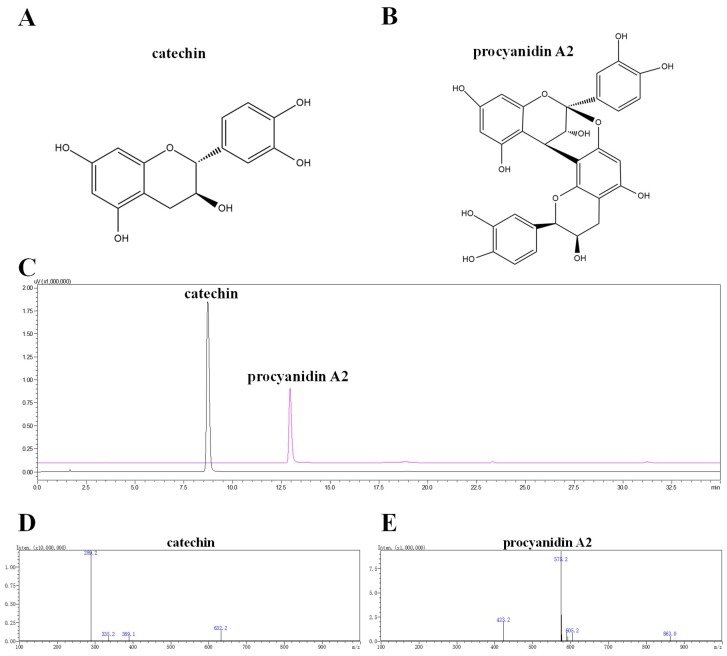
UHPLC-DAD-ESI-MS/MS chromatograms of catechin and procyanidin A2. The structures of (**A**) catechin and (**B**) procyanidin A2. (**C**) DAD chromatograms of catechin and procyanidin A2 recorded at 280 nm, MS spectrum of (**D**) catechin and (**E**) procyanidin A2 in negative mode.

LSF was prepared according to the previous study [19,20] and first analyzed using RP-HPLC monitored at 280 nm. A total of 12 main peaks were collected using AKTA protein purification system (Figure S1A,B). The purity of 12 collected peaks were measured by using RP-HPLC (Figure S1C). Through the preliminary evaluation of the anti-neuroinflammation effect of these 12 fractions using Wright–Giemsa staining and ELISA methods, Fractions 3 and 9 were found to improve the morphological status, decrease the ratio of activated BV-2 cells, increase cell density, and decrease the release of pro-inflammatory cytokines in Aβ(1-42)-induced BV-2 cells (Figures S2 and S3), suggesting that the compounds in Fraction 3 and 9 could be the major bioactive components that are responsible for the anti-inflammation effect. Therefore, we applied pre-HPLC to further purify the compounds in Fractions 3 and 9, and obtained compounds **1** and **2** from Fractions 3 and 9, respectively. Through the elucidation using ultra-high-performance liquid chromatography with diode array detector coupled with electrospray ionization-tandem mass spectrometry (UHPLC-DAD-ESI-/MS/MS) and NMR instruments (Figures S4–S6), Compounds **1** and **2** were confirmed to be catechin and procyanidin A2, respectively. Their structures, DAD chromatograms, and MS spectrums are displayed in Figure 1 and Figure S7.

Compound **1**: UV λmax: 215, 276 nm, LC-ESI-MS/MS: *m/z* 289.2 [M − H]^−^, ^1^H-NMR (400 MHz, DMSO): d 9.22 (s, 1H, 5-OH), 8.97 (s, 1H, 7-OH), 8.90 (s, 1H, 3′-OH), 8.85 (s, 1H, 4′-OH), 6.71 (m, 1H, 2′-H), 6.67 (d, *J* = 1.9 Hz, 1H, 5′-H), 6.60 (d, *J* = 1.9 Hz, 1H, 6′-H), 5.88 (d, *J* = 2.3 Hz, 1H, 6-H), 5.68 (d, *J* = 2.2 Hz, 1H, 8-H), 4.90 (d, *J* = 5.1 Hz, 1H, 3-OH), 4.48 (d, *J* = 7.5 Hz, 1H, 2-H), 3.81 (m, 1H, 3-H), 2.63 (dd, *J* = 16.1, 5.3 Hz, 1H, 4-H), 2.35 (dd, *J* = 16.0, 8.0 Hz, 1H, 4-H); ^13^C-NMR (101 MHz, DMSO): d 156.90 (C-7), 156.62 (C-5), 155.80 (C-8a), 145.28 (C-39, 49), 131.00 (C-19), 118.88 (C-69), 115.49 (C-29), 114.94 (C-59), 99.47 (C-4a), 95.49 (C-6), 94.24 (C-8), 81.44 (C-2), 66.73 (C-3), 28.34 (C-4). The above data were consistent with the reported papers and identified as catechin [21,22].

Compound **2**: UV λmax: 215, 276 nm, LC-MS/MS: *m/z* 575.2 [M − H]^−^, ^1^H-NMR (400 MHz, MeOD) δ 7.14 (1H, d, *J* = 2.0 Hz, H-2″), 7.13 (1H, d, *J* = 2.0 Hz, H-2′″), 7.01 (1H, dd, *J* = 8.3, 2.2 Hz, H-6″), 6.97 (1H, dd, *J* = 8.3, 2.0 Hz, H-6′″), 6.81 (1H, d, *J* = 1.7 Hz, H-5″), 6.79 (1H, d, *J* = 1.8 Hz, H-5′″), 6.08 (1H, s, H-6′), 6.06 (1H, d, *J* = 2.3 Hz, H-8), 5.99 (1H, d, *J* = 2.4 Hz, H-6), 4.92 (1H, s, H-2′), 4.40 (1H, d, *J* = 3.4 Hz, H-4), 4.23 (1H, m, H-3′), 4.05 (1H, d, *J* = 3.4 Hz, H-3), 2.94 (1H, dd, *J* = 17.2, 4.9 Hz, H-4′α), 2.75 (1H, dd, *J* = 17.2, 2.3 Hz, H-4′β); ^13^C-NMR (101 MHz, MeOD) δ 158.29 (C-7), 157.15 (C-5), 156.76 (C-5′), 154.42 (C-9), 152.46 (C-7′), 152.30 (C-9′), 146.93 (C-3′″), 146.47 (C-3″), 146.16 (C-4′″), 145.82 (C-4″), 132.63 (C-1″), 131.36 (C-1′″), 120.57 (C-6″), 119.97 (C-6′″), 116.23 (C-5″), 116.1 (C-5′″), 115.85 (C-2″), 115.82 (C-2′″), 107.39 (C-8′), 104.45 (C-10), 102.62 (C-10′), 100.36 (C-2), 98.50 (C-6), 96.82 (C-8), 96.70 (C-6′), 81.94 (C-2′), 68.24 (C-3), 67.14 (C-3′), 30.06 (C-4′), 29.43 (C-4). The above data were consistent with the reported papers and identified as procyanidin A2 [23].

### 2.2. Catechin and Procyanidin A2 Improve the Morphology of BV-2 Cells

**Figure 2:**
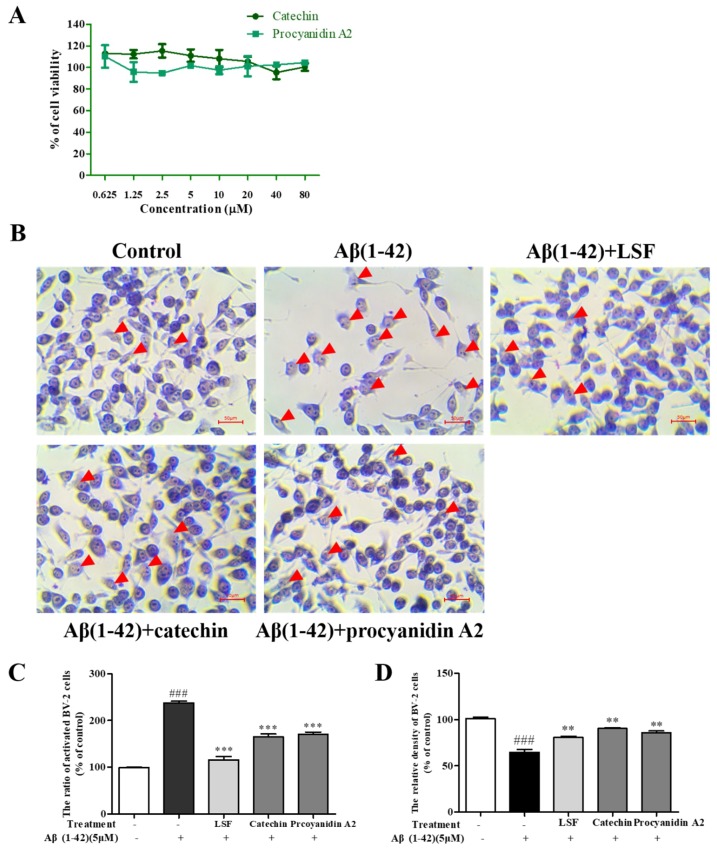
Morphological changes of BV-2 cells. (**A**) Cytotoxicity of catechin and procyanidin A2 against BV-2 cells for 48 h using CCK-8 kit; (**B**) BV-2 cells were pretreated with 5 µM Aβ(1-42) for 12 h, followed by incubations of LSF (0.469 µg/mL), catechin (10 µM), and procyanidin A2 (10 µM), respectively. The red arrows indicate the activated BV-2 cells with ameboid shape. Magnification: ×100; Scale bar: 50 µm. (**C**) The ratio of activated BV-2 cells, which was calculated by counting the number of activated BV-2 cells in all of the BV-2 cells views; (**D**) The density of BV-2 cells, which was quantified by counting the number of all the BV-2 cells, and a minimum of 150 cells from three randomly selected fields were scored. BV-2 cells treated with medium alone were set as the control group, and BV-2 cells treated with 5 µM Aβ(1-42) alone were set as the Aβ group. ^###^ *p* < 0.001 vs. Control group; ** *p* < 0.005, *** *p* < 0.001 vs. Aβ group.

The cellular morphology of BV-2 cells can reflect the inflammatory response [24]. In this part, we first examined the cytotoxicity of catechin and procyanidin A2 against BV-2 cells using a CCK-8 kit, and there no cytotoxicity was observed under 80 µM (Figure 2A). Figure 2B showed the morphologic changes of BV-2 cells treated with Aβ(1-42) alone and Aβ(1-42) co-treated with LSF, catechin, and procyanidin A2, which were detected by the Wright–Giemsa staining method. As shown in Figure 2B–D, LSF, catechin and procyanidin A2 could reduce the ratio of activated BV-2 cells that displayed ameboid shape, fusiform, more synapse and obvious nucleus fragmentations, and increase cell density in Aβ(1-42)-induced BV-2 cells, suggesting that LSF and its derived compounds could suppress Aβ(1-42) induced neuroinflammation. Therefore, catechin and procyanidin A2 could be the bioactive components that are responsible for the anti-neuroinflammation effect.

### 2.3. Catechin and Procyanidin A2 Inhibit Pro-Inflammatory Cytokines in Aβ(1-42)-Induced BV-2 Cells

**Figure 3:**
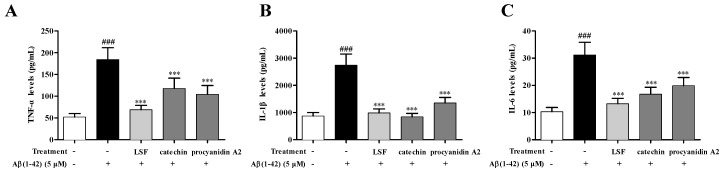
The release of TNF-α, IL-1β, and IL-6 in Aβ(1-42)-induced BV-2 cells under the treatments of LSF, catechin, and procyanidin A2. BV-2 cells were pretreated with 5 µM Aβ(1-42) for 12 h, followed by incubations of LSF (0.469 µg/mL), catechin (10 µM), and procyanidin A2 (10 µM) for another 12 h. After treatment, the cell free supernatants were subsequently employed for TNF-α (**A**), IL-1β (**B**), and IL-6 (**C**) assays using ELISA kit. BV-2 cells treated with medium alone were set as the control group, and BV-2 cells treated with 5 µM Aβ(1-42) alone were set as the Aβ group. ^###^ *p* < 0.001 vs. control group; *** *p* < 0.001 vs. Aβ group. Data are the mean value ± S.D. of ten independent experiments.

**Figure 4:**
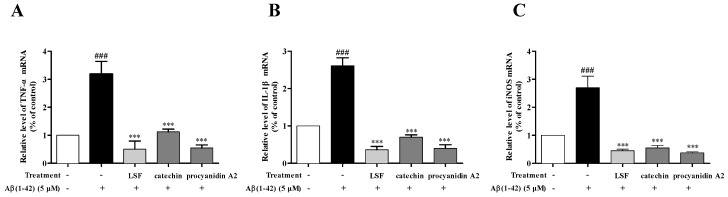
The mRNA levels of TNF-α, IL-1β, and iNOS in Aβ(1-42)-induced BV-2 cells under the treatments of LSF, catechin, and procyanidin A2. BV-2 cells were pretreated with 5 µM Aβ(1-42) for 12 h, followed by incubations of LSF (0.469 µg/mL), catechin (10 µM), and procyanidin A2 (10 µM) for another 12 h. After treatment, total mRNA were extracted and subsequently employed for TNF-α (**A**), IL-1β (**B**), and iNOS (**C**) measurements using RT-PCR. BV-2 cells treated with medium alone were set as the control group, and BV-2 cells treated with 5 µM Aβ(1-42) alone were set as the Aβ(1-42) group. ^###^ *p* < 0.001 vs. control group; *** *p* < 0.001 vs. Aβ group. Data are the mean value ± S.D. of five independent experiments.

**Figure 5:**
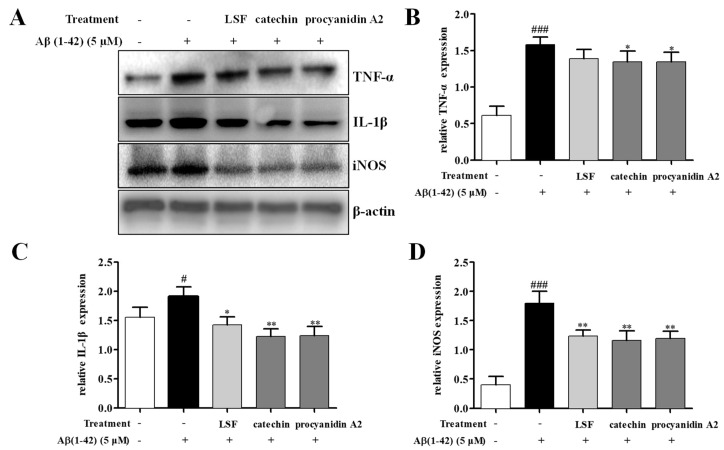
The protein expressions of TNF-α, IL-1β, and iNOS in Aβ(1-42)-induced BV-2 cells under the treatments of LSF (0.469 µg/mL), catechin (10 µM), and procyanidin A2 (10 µM). BV-2 cells were pretreated with 5 µM Aβ(1-42) for 12 h, followed by incubations of LSF, catechin, and procyanidin A2 for another 12 h. (**A**) After treatment, cell lysates were harvested and subsequently employed for TNF-α, IL-1β, and iNOS measurements using Western blotting. Band intensities of TNF-α (**B**), IL-1β (**C**), and iNOS (**D**) were quantified using Image J software and normalized to β-actin. Bars are representatives of three independent experiments. BV-2 cells treated with medium alone were set as the control group, and BV-2 cells treated with 5 µM Aβ(1-42) alone were set as the Aβ group. ^#^ *p* < 0.05, ^###^ *p* < 0.001 vs. control group; * *p* < 0.05, ** *p* < 0.01 vs. Aβ group. The full-length Western blotting images are shown in Figure S8.

Our previous study proved that LSF could inhibit the release, mRNA levels, and protein expressions of TNF-α, IL-1β, COX-2, and iNOS. Based on the preliminary screen result of the anti-neuroinflammation effect of the 12 isolated fractions, we further validated the anti-neuroinflammation effect of catechin and procyanidin A2 in Fractions 3 and 9 in the current experiment. As shown in Figure 3, LSF, catechin and procyanidin A2 could significantly decrease the release of TNF-α, IL-1β, and IL-6 levels in cell culture supernatants using an ELISA kit in Aβ(1-42)-induced BV-2 cells. Additionally, the mRNA levels of TNF-α, IL-1β, and iNOS were measured using RT-PCR. Figure 4 shows that LSF, catechin, and procyanidin A2 could significantly reduce the mRNA levels in Aβ(1-42)-induced BV-2 cells. Correspondingly, the protein expressions of TNF-α, IL-1β, and iNOS in Aβ(1-42)-induced BV-2 cells under the treatments of LSF, catechin, and procyanidin A2 were detected by Western blotting. As shown in Figure 5, LSF, catechin, and procyanidin A2 dramatically reduced the protein content of TNF-α, IL-1β, and iNOS. In summary, catechin and procyanidin A2 could inhibit the inflammatory response in Aβ(1-42)-induced BV-2 cells, suggesting that catechin and procyanidin A2 could be the key active components in LSF.

### 2.4. Catechin and Procyanidin A2 Inhibit the Activation of NF-κB Signaling Pathway

**Figure 6:**
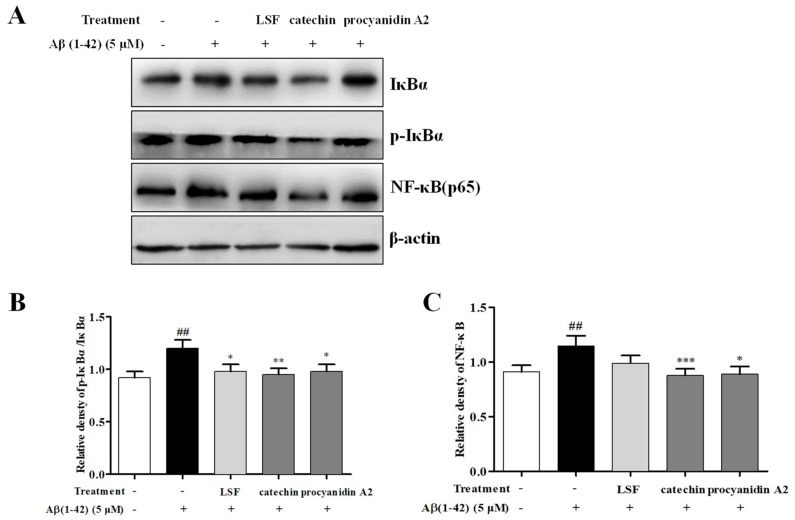
Catechin and procyanidin A2 inhibit the activation of the NF-κB signaling pathway. BV-2 cells were pretreated with 5 µM Aβ(1-42) for 12 h, followed by incubations of LSF (0.469 µg/mL), catechin (10 µM), and procyanidin A2 (10 µM) for another 12 h. (**A**) After treatment, the cell lysates were harvested and subsequently employed for IκBα, p-IκBα, and NF-κB detections using Western blotting. Band intensities of p-IκBα/IκBα (**B**) and NF-κB (**C**) were quantified using Image J software and normalized to β-actin. Bars are representatives of three independent experiments. BV-2 cells treated with medium alone were set as the control group, and BV-2 cells treated with 5 µM Aβ(1-42) alone were set as the Aβ group. ^##^ *p* < 0.01 vs. control group; * *p* < 0.05, ** *p* < 0.01, *** *p* < 0.001 vs. Aβ group. The full-length Western blotting images are shown in Figure S8.

Emerging evidence implies that the NF-κB signaling pathway is activated in AD pathogenesis. This pathway can regulate the release and expression of pro-inflammatory cytokines, and its function is related to its inhibitory factor, IκBα. Aβ can activate the NF-κB signaling pathway then release the pro-inflammatory cytokines. In this study, we investigated the inhibition effect of LSF and the isolated components, catechin and procyanidin A2 in neuroinflammation in Aβ(1-42)-induced BV-2 cells. As shown in Figure 6, LSF, catechin, and procyanidin A2 could significantly suppress the protein expression of NF-κB and the ratio of p-IκBα/IκBα, suggesting that LSF, catechin, and procyanidin A2 suppressed the neuroinflammation via inhibiting the NF-κB signaling pathway in Aβ(1-42)-induced BV-2 cells.

### 2.5. Catechin and Procyanidin A2 Inhibit Cell Apoptosis in Aβ(1-42)-Induced BV-2 Cells

**Figure 7:**
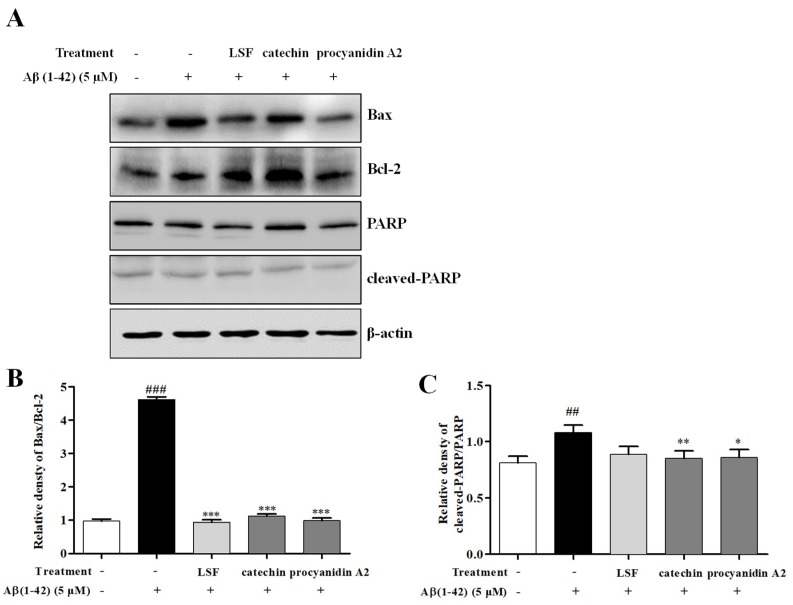
Catechin and procyanidin A2 inhibit cell apoptosis in the Aβ(1-42)-induced BV-2 cells. BV-2 cells were pretreated with 5 µM Aβ(1-42) for 12 h, followed by incubations of LSF (0.469 µg/mL), catechin (10 µM), and procyanidin A2 (10 µM) for another 12 h. (**A**) After treatment, cell lysates were harvested and subsequently employed for Bax, Bcl-2 PARP, and cleaved-PARP detections using Western blotting. Band intensities of Bax/Bcl-2 (**B**) and cleaved-PARP/PARP (**C**) were quantified using Image J software and normalized to β-actin. Bars are representatives of three independent experiments. BV-2 cells treated with medium alone were set as the control group, and BV-2 cells treated with 5 µM Aβ(1-42) alone were set as the Aβ group. ^##^ *p* < 0.01, ^###^ *p* < 0.001 vs. control group; * *p* < 0.05, ** *p* < 0.01, *** *p* < 0.001 vs. Aβ group. The full-length Western blotting images are shown in Figure S8.

As is known to us, over-expression of pro-inflammatory cytokines can induce the microglial activation and lead to neuronal death. Our previous study have proved that LSF could inhibit cell apoptosis in Aβ(1-42)-induced BV-2 cells. In this current experiment, we also investigated the suppression effect of cell apoptosis in Aβ(1-42)-induced BV-2 cells under the treatments of LSF and the isolated components, catechin and procyanidin A2. As shown in Figure 7, LSF, catechin and procyanidin A2 could inhibit Aβ(1-42)-induced BV-2 cells apoptosis by reducing Bax/Bcl-2 ratio and attenuating the proportion of cleaved-PARP in total PARP. These results suggested that the decreased cell apoptosis could relate to the inhibition of inflammatory response in Aβ(1-42)-induced BV-2 cells with the treatments of catechin and procyanidins A2.

## 3. Discussion

AD is the most common type of dementia worldwide. Its pathological hallmarks are recognized as the accumulated Aβ in SPs and the hyperphosphorylation of tau in NFTs [25,26]. The inactivated microglia can improve Aβ clearance and promote anti-inflammatory effects, and also protect the CNS from injury and invading pathogens [27]. However, it has recently been shown that microglial depletion for 2–4 weeks in the mouse model at advanced stage cannot reduce Aβ plaque, suggesting that the aged microglia is inactivated in clearing plaques in the AD brain [28]. It has been reported that immune activation early in life could trigger the onset of AD, while exposing microglia to Aβ could induce sustained chronic neuroinflammation, tau hyperphosphorylation, and a loss of synapses and neurons [7,29,30,31]. Therefore, the timing of Aβ pathogenesis is recognized to be critical for microglia to form a physical barrier to limit neurotoxicity [32], and a potent inhibitor of microglia activation is essential for inhibiting neuroinflammation. Lychee seed, a commonly used TCM, has been reported to exert many biological activities such as anti-oxidation, anti-virus, and improving insulin resistance (IR) [33,34,35,36]. In our previous study, LSF, a bioactive fraction from lychee seed, was first proven to improve the cognitive function of AD rats, protect PC12 cells against Aβ-induced injury, and suppress neuroinflammation in Aβ(1-42)-induced BV-2 cells [19,20]. However, the specific components being responsible for this effect are still unknown. In this experiment, we isolated and identified two bioactive components from LSF that are responsible for these neuroprotective effects.

It has been reported that SP is surrounded by a cluster of reactive microglia as phagocytic cells. These microglia can remove Aβ in brain and protect neurons [29,37,38]. On the other hand, studies have shown that the content of Aβ in microglia was remarkably increased in AD patients and model animals’ brains, suggesting that the microglia are over activated and then contribute to the occurrence of AD [39,40]. Therefore, the over-activated microglia induced by accumulated Aβ or tau will induce neuroinflammation and accelerate AD progression. Based on this evidence, we used Aβ(1-42)-induced BV-2 cells to simulate the over activated microglia in AD patients or rats. With persistent activation of BV-2 cells by Aβ(1-42), many cytotoxic factors including TNF-α, IL-1β, IL-6, nitric oxide (NO), and superoxide are released from the over-activated microglia [29,41,42,43]. The released toxic inflammatory cytokines induce microglial activation and lead to neuronal death, and ultimately dementia [44]. In the present study, we symmetrically isolated and identified two polyphenols including catechin and procyanidin A2 from LSF using column chromatography, pre-HPLC, and NMR technologies. The bioactive validation proved that catechin and procyanidin A2 could suppress Aβ-induced neuroinflammation in Aβ(1-42)-induced BV-2 cells by inhibiting the release, mRNA levels, and protein expressions of TNF-α, IL-1β, IL-6, and iNOS.

Polyphenols widely exist in most natural plants such as *Radix et Rhizoma Rhei*, *Radix Scutellariae*, *Rhizoma et Radix Polygoni Cuspidati*, etc. As is known to us, polyphenols exert a potent anti-oxidative effect and protect cells or tissue against oxidative damage, which results in improved immunity, anti-cancer, and anti-aging effects. Recently, many polyphenols such as resveratrol from *Rhizoma et Radix Polygoni Cuspidati* [45]; epigallocatechin gallate (EGCG) from green tea [46]; curcumin from the spice turmeric [47]; and the flavonoids—including baicalin, baicalein, wogonoside, wogonin, oroxylin A, etc.—from *Radix Scutellariae* [48] have been proved to inhibit Aβ aggregation. Catechin and procyanidin A2 widely exist in fruit seed and skin including grape seed, apple seed, and the skin from *Hylocereus undatus ‘Foo-Lon’*. Catechin has been reported to stimulate antioxidant activity by scavenging free radicals, inhibiting pro-oxidant enzymes, and stimulating antioxidant enzymes. In this study, our data suggested that catechin inhibited neuroinflammation, which is consistent with previous studies that claimed that catechin possessed a neuroprotective effect [49]. Procyanidins are members of the proanthocyanidin (or condensed tannins) class of flavonoids. They are oligomeric compounds composed of catechin and epicatechin. It has been reported that procyanidins have an extremely strong antioxidant and free radical clearing effect. At the same time, catechin and procyanidins have also been proved to inhibit Aβ-induced apoptosis in PC12 cells and inhibit Aβ aggregation, respectively [50,51]. However, up to now, there have been no reports on the inhibition effect of catechin and procyanidin A2 in Aβ-induced neuroinflammation.

In this study, we identified the key active components of LSF to inhibit Aβ(1-42)-mediated neuroinflammation. In general, morphological appearance is often related to functional properties, and we found the appearance of BV-2 cells treated with Aβ(1-42) were ameboid shaped, indicating a fully activated state [52,53]. Furthermore, the proportion of ameboid-shaped cells in Aβ(1-42) pre-treated BV-2 cells was decreased after the treatment of LSF, catechin, or procyanidin A2, suggesting that LSF, catechin, and procyanidin A2 can inhibit BV-2 cells activation.

NF-κB is a protein complex that regulates the transcription of DNA, pro-inflammatory cytokine release, and cell survival [54,55,56,57,58]. Pro-inflammatory mediators such as TNF-α, IL-1, IL-6, iNOS, and cyclooxygenase-2 (COX-2) are released from cells as NF-κB is activated [59]. NF-κB usually exists in the cytoplasm in the form of homo- and/or heterodimers, and forms an inactive complex with its inhibitory factor IκBα [60,61]. IκBα is phosphorylated by IκB kinase (IKK) and releases the NF-κB dimer when the pathway is activated. Our previous studies proved that LSF could inhibit neuronal and BV-2 cells apoptosis, which could be associated with the inhibition of the NF-κB pathways [20]. In the current study, catechin and procyanidin A2 in LSF were found to inhibit the NF-κB pathway by downregulating the p-IκBα/IκBα and NF-κB expressions.

Aβ also induces microglial apoptosis and further aggravates the inflammatory response. The Bcl-2 family plays an important role in the regulation of apoptosis. It consists of anti-apoptotic proteins such as Bcl-2, Bcl-xl, Bcl-w, and Mcl-1, and pro-apoptotic proteins including Bax, Bak, Bad, Bid, and Bim. In the present study, we found that catechin and procyanidin A2 could decrease the Bax/Bcl-2 ratio in Aβ(1-42)-induced BV-2 cells. Additionally, another apoptosis related protein, the poly (ADP-ribose) polymerase (PARP) protein family, is associated with many cellular processes such as DNA repair, genomic stability, and programmed cell death. The cleaved-PARP is generally considered to be an important indicator of apoptosis and caspase 3 activation [62,63]. Similarly, catechin and procyanidin A2 could reduce the proportion of cleaved-PARP in total PRAP in Aβ(1-42)-induced BV-2 cells. Taken together, all of the above results indicated that catechin and procyanidin A2 could suppress apoptosis in Aβ-induced BV-2 cells.

The blood–brain barrier (BBB) permeability of the drugs acting on CNS is becoming an important issue to which we should pay attention. The permeability of dietary polyphenols across the blood–brain barrier (BBB) is selective, has poor absorption, and rapid metabolism, which limits their bioavailability and neuroprotective effect. Most polyphenols have been proven to have a neuroprotective effect in vitro and vivo. Recently, a study found that the bioavailable polyphenol metabolites could transport across the BBB endothelium and protect brain endothelial cells and neuronal cells, and reduce neuroinflammation [64]. Although the polyphenols intracellular concentrations in neurons and microglial cells are limited, such concentrations are in fact beneficial [65]. Catechin, the compound identified from LSF in the current study, has previously been reported to pass through BBB after intravenous (iv) administration [66]. In addition, nanosystems could be a promising strategy for delivering resveratrol, a polyphenol, into the brain, which can protect it from degradation in the blood stream [67]. Therefore, a rational administration or formulation could avoid the first pass effect, protect degradation, and improve the BBB permeability of the polyphenols. 

Collectively, our study confirmed that the two key active substances of LSF, catechin and procyanidin A2, could inhibit Aβ-induced neuroinflammation. In future study, we will try to investigate the concentration of catechin and procyanidin A2 passed through BBB by selecting the optimal administration route and formulation, which are expected to be developed as novel drugs for AD.

## 4. Materials and Methods

### 4.1. Reagent and Instrument

LSF was prepared according to our previous method [19,20]. HPLC-grade acetonitrile and methanol, sodium dodecyl sulfate (SDS), and Aβ(1-42) were purchased from Sigma-Aldrich (St. Louis, MO, USA). Roswell Park Memorial Institute 1640 medium (RPMI 1640) was obtained from Gibco (Grand Island, NY, USA). Fetal bovine serum (FBS), RT-PCR kit, and PCR kit were purchased from TransGen Biotech Inc. (Beijing, China). The Wright–Giemsa stain kit was purchased from Nanjing Jiancheng Bioengineering Institute (Nanjing, China). Primary antibodies such as TNF-α, IL-1β, inducible nitric oxide synthase (iNOS), nuclear factor kappa-light-chain-enhancer of activated B (NF-κB), inhibitor of NF-κB alpha (IκBα), phosphorylate-IκBα (p-IκBα), poly ADP-ribose polymerase (PARP), cleaved-PARP, Bax, Bcl-2, and β-actin were obtained from Cell Signaling Technology (CST) (Beverly, MA, USA). Enzyme linked immunosorbent assay (ELISA) kits of TNF-α, IL-1β, and IL-6 were purchased from Beijing Cheng Lin Biological Technology Co., Ltd. (Beijing, China).

### 4.2. Instrument and Chromatograph Condition for the Separation of the Components in LSF

The separation and purification of the components in LSF was performed using AKAT protein purification system equipped with a UV–vis detector at 280 nm on an amethyst C_18_–H column (21.2 × 250 mm, 5 μm) which was purchased from Sepax Technologies Inc. (Suzhou, China) at a flow rate of 10 mL/min. The parameters of the gradient elution program were applied as follows: the ratio of mobile phase B (acetonitrile) to mobile phase A (0.1% formic acid in water) changed from 15% to 35% in 45 min. The peaks displaying in the chromatograms were collected according to their retention time. All the collected solutions were concentrated by rotary evaporator.

### 4.3. Cell Culture

BV-2 cells, an immortalized mouse microglia cell line, was purchased from the Kunming Institute of Zoology, Chinese Academy of Science (Kunming, China). It was cultured in RPMI 1640 medium supplemented with 20% fetal bovine serum and 1% penicillin/streptomycin at 37 °C with CO_2_ (5%) [8].

### 4.4. Cell Viability

Twelve isolated fractions were dissolved with dimethyl sulfoxide (DMSO) to make a final concentration of 80 g/L. Cell viability of Fractions 1–12 against BV-2 cells was measured with a CCK-8 kit according to the manufacture’s protocols. In brief, BV-2 cells were plated on 96-well plates (100 µL, 1 × 10^5^ cells/well) one day before LSF treatment under the indicated concentrations. After 48 h treatment, 10 µL of CCK-8 solution was added into each well and incubated for another 2 h. Colorimetric reading of the solute mixture was then determined at OD 450 nm using a standard plate-reader (DG5032, Hua Dong, Nanjing, China). The percentage of cell viability was calculated using the following formula: Cell viability (%) = Cells number _treated_/Cells number _DMSO control_ × 100. Data were obtained from three independent experiments.

### 4.5. Identification of the Components in LSF

Reverse phase ultra-high-performance liquid chromatography (RP-UHPLC) (Shimadzu Corporation, Hadano, Japan) equipped with DGU-20A5R degasser, two LC-20ADXR pumps, a SIL-20ACXR autosampler, a CTO-20AC column oven, a SPD-M20A diode array detector, and a Shimadzu CBM-20A system controller was used for analysis in the current study. All the samples dissolved in methanol were separated by a C18 column (4.6 × 250 mm, 5.0 μm particle size) (GL Science, Torrance, CA, USA) at a flow rate of 1 mL/min. The gradient elution system consisting of mobile phase A (acetonitrile) and mobile phase B (0.1% acetic acid, *v*/*v*) was set as follows: 0–10 min, 5–24% (A); 10–11 min, 24–24% (A); 11–11.01 min, 24–28% (A); 11.01–12 min, 28–28% (A); 12–12.01 min, 28–36% (A); 12.01–13 min, 36–36% (A); 13–13.01 min, 36–40% (A); 13.01–14 min, 40–40% (A); 14–14.01 min, 40–45% (A); 14.01–15 min, 55–55% (A); 15–15.01 min, 55–50% (A); 15.01–16 min, 50–50% (A); 16–18 min, 50–60% (A); 18–30 min, 60–70% (A). The column temperature was maintained at 25 °C and the injection volume was 20 μL of each sample. The UV–vis absorption spectra were recorded on-line from 190 to 800 nm during the analysis and the components in LSF were monitored at 280 nm. Data analysis was carried out using Shimadzu LabSolutions software B.01.03 (Shimadzu Corporation, Hadano, Japan).

Ultra-high-performance liquid chromatography (UHPLC) (Shimadzu Corporation, Hadano, Japan) equipped with DGU-30A5R degasser, two LC-30ADXR pumps, a SIL-30ACXR autosampler, a CTO-30AC column oven, a SPD-M30A diode array detector, a Shimadzu CBM-30A system controller, and a 8045 electrospray ionization tandem mass spectroscopic (ESI-MS/MS) was used to identify the isolated components from LSF in negative and positive modes. Data were acquired in scan mode from *m*/*z* 100 to 1000 Da with 2.0 spectra/s. Data analysis was carried out using Shimadzu LabSolutions software B.01.03 (Shimadzu Corporation, Hadano, Japan).

### 4.6. Wright–Giemsa Staining

BV-2 cells were seeded on coverslips in a 6-well plate a day before treatments. The cells were pretreated with 5 µM Aβ(1-42) for 12 h, followed by incubations of the test fractions or compounds for another 12 h. After treatments, these coverslips were air-dried and stained with Giemsa solution as described previously [68]. In brief, slides stained in the diluted Giemsa solution (1:20) in PBS for 15–20 min were washed with PBS to remove the excess stain and air-dried at room temperature. The air-dried slides were mounted with FluorSave^TM^ mounting media (Calbiochem, San Diego, CA, USA), and the optical microscope images were observed and taken with an optical microscope (Leica DM750 optical microscope, Leica, Germany). The ratio of activated BC-2 cells was calculated by counting the number of the activated BV-2 cells in all of the BV-2 cells. In addition, the density of BV-2 cells was quantified by counting the number of all of the BV-2 cells. A minimum of 150 cells from three randomly selected fields were scored.

### 4.7. Cytokines ELISA

BV-2 cells were plated on a 96-well plate (100 µL, 1 × 10^5^ cells/well) a day before treatments. The cells were pretreated with 5 µM Aβ(1-42) for 12 h, followed by an incubation of the fractions or compounds for another 12 h. The cell-free supernatants were subsequently employed for TNF-α, IL-1β, and IL-6 assays using the ELISA kits according to the manufacturer’s instructions.

### 4.8. Quantitative Reverse Transcription PCR (qRT-PCR)

The total RNA of BV-2 cells was extracted by TRIZOL reagent, and its reverse transcription into cDNA were performed according to the manufacture’s protocol for the Prime Script RT reagent kit (Trans Serum, Beijing, China). The primers are listed as follows
TNF-αForward: 5′-GAGCACAGAAAGCATGATCC-3′Reverse: 5′-GAGAAGAGGCTGAGACATAG-3′IL-1βForward: 5′-CTAGGGACTTAGGTGCTGTC-3′Reverse: 5′-CTCTGCCTTTGCTTCCAAGC-3′iNOSForward: 5′-CGTTGGATTTGGAGCAGAAG-3′Reverse: 5′-CCTCTTTCAGGTCACTTTGG-3′GAPDHForward: 5′-GACAGTCGGAAACTGGGAAG-3′Reverse: 5′-CATCACGTCCTCCATCATCC-3′

### 4.9. Western Blotting

After treatment, cells were washed with pre-cooled PBS and lysed by RIPA lysis buffer (Beyotime, Shanghai, China) containing 20 mM Tris-HCl (pH 7.4), 1% Triton X-100, 140 mM NaCl, and 1 mM phenylmethylsulfonyl fluoride (PMSE). Protein concentrations were measured by BCA kit (Beyotime, Shanghai, China) as per the previous report [20]. Equal amounts of each protein (50 µg/well) were loaded onto SDS-PAGE and the separated proteins on the gel were transferred onto a polyvinylidene difluoride (PVDF) membrane, which was then blocked with 5% non-fat milk in Tris-buffered saline and Tween 20 (TBST) for 1 h. The membrane was then washed with TBST three times and incubated with the primary antibodies including β-actin, TNF-α, IL-1β, iNOS, NF-κB, IκBα, p-IκBα, PARP, cleaved-PARP, Bax, and Bcl-2 overnight at 4 °C. After the membrane was washed with TBST three times, it was followed with a further incubation for 1 h at room temperature with horseradish peroxidase (HRP) conjugated secondary antibodies. The protein expression bands were revealed by the ultra ECL Detection Reagent ECL (4A Biotech Co., Ltd., Beijing, China) and detected by the ChemiDoc MP Imaging System (Bio-Rad, Hercules, CA, USA). Band intensity was quantified using Image J software (National Institutes of Health, Bethesda, MD, USA) and the ratio of the interest proteins to β-actin was calculated.

### 4.10. Statistical Analysis

All data were presented as the mean ± standard deviation (S.D.) and were analyzed using GraphPad Prism 5.0 statistical analytical software (GraphPad Software, San Diego, CA, USA). The data were considered to have significant difference as *p* < 0.05. One-way ANOVA followed by the post-Tukey test was applied for statistical analysis to compare all the different groups in the current study.

## 5. Conclusions

In the current study, two polyphenols—catechin and procyanidin A2—were isolated and identified in LSF derived from lychee seed; they were proven to be the bioactive components responsible for the inhibition effect of neuroinflammation in Aβ(1-42)-induced BV-2 cells by modulating the NF-κB signaling pathway. Furthermore, catechin and procyanidin A2 could also inhibit cell apoptosis in Aβ(1-42)-induced BV-2 cells. This study illuminated the action substances and molecular mechanism of LSF in anti-neuroinflammation. Further studies on brain pharmacokinetics and the pharmacological activity in vivo are also essential.

## Supplementary Materials

Supplementary materials can be found at http://www.mdpi.com/1422-0067/19/7/2109/s1.

## Abbreviations

Aβ	Amyloid-β
AD	Alzheimer’s disease
LSF	Lychee seed fraction
pre-HPLC	Preparation of high performance liquid chromatography
NMR	Nuclear magnetic resonance
TNF-α	Tumor necrosis factor-α
IL-1β	Interleukin-1β
IL-6	Interleukin-6
NF-κB	Nuclear factor-κB
ELISA	Enzyme linked immunosorbent assay
RT-PCR	Real time-PCR
SPs	Senile plaques
NFTs	Neurofibrillary tangles
CNS	Central nervous system
PD	Parkinson’s disease
HD	Huntington’s disease
NO	Nitric oxide
ROS	Reactive oxygen species
TCMs	Traditional Chinese medicines
T2DM	Type 2 diabetes mellitus
COX-2	Cyclooxygenase 2
iNOS	Inducible nitric oxide synthase
IκBα	NF-κB inhibitor α
PARP	Poly ADP-ribose polymerase
IR	Insulin resistance
EGCG	Epigallocatechin gallate
IKK	IκB kinase
BBB	Blood–brain barrier

## References

[1] Wang X., Wang W., Li L., Perry G., Lee H., Zhu X. (2014). Oxidative stress and mitochondrial dysfunction in Alzheimer’s disease. BBA Mol. Basis Dis..

[2] Scheff S.W., Price D.A., Schmitt F.A., Mufson E.J. (2006). Hippocampal synaptic loss in early Alzheimer’s disease and mild cognitive impairment. Neurobiol. Aging.

[3] Varnum M.M., Ikezu T. (2012). The Classification of Microglial Activation Phenotypes on Neurodegeneration and Regeneration in Alzheimer’s Disease Brain. Arch. Immunol. Ther. Exp..

[4] Heneka M.T., Carson M.J., Khoury J.E., Landreth G.E. (2015). Neuroinflammation in Alzheimer’s disease. Lancet Neurol..

[5] Ferreira S.T., Clarke J.R., Bomfim T.R., de Felice F.G. (2014). Inflammation, defective insulin signaling, and neuronal dysfunction in Alzheimer’s disease. Alzheimers Dement..

[6] Hung A.S.M., Liang Y., Chow T.C.H., Tang H.C., Wu S.L.Y., Wai M.S.M., Yew D.T. (2016). Mutated tau, amyloid and neuroinflammation in Alzheimer disease—A brief review. Prog. Histochem. Cytochem..

[7] Streit W.J., Mrak R.E., Griffin W.S. (2004). Microglia and neuroinflammation: A pathological perspective. J. Neuroinflamm..

[8] Yuan L., Wu Y., Ren X., Liu Q., Wang J., Liu X. (2014). Isoorientin attenuates lipopolysaccharide-induced pro-inflammatory responses through down-regulation of ROS-related MAPK/NF-κB signaling pathway in BV-2 microglia. Mol. Cell. Biochem..

[9] Song S., Jung Y.Y., Hwang C.J., Lee H.P., Sok C.H., Kim J.H., Lee S.M., Seo H.O., Hyun B.K., Choi D.Y. (2014). Inhibitory effect of ent-Sauchinone on amyloidogenesis via inhibition of STAT3-mediated NF-κB activation in cultured astrocytes and microglial BV-2 cells. J. Neuroinflamm..

[10] Bhaskar K., Maphis N., Xu G., Varvel N.H., Kokiko-Cochran O.N., Weick J.P., Staugaitis S.M., Cardona A., Ransohoff R.M., Herrup K. (2014). Microglial derived tumor necrosis factor-α drives Alzheimer’s disease-related neuronal cell cycle events. Neurobiol. Dis..

[11] Beauchet O., Launay C.P., Allali G., Annweiler C. (2014). Changes in Gait Variability with Anti-dementia Drugs: A Systematic Review and Meta-analysis. CNS Drugs.

[12] Wei S. (2016). Potential therapeutic action of natural products from traditional Chinese medicine on Alzheimer’s disease animal models targeting neurotrophic factors. Fund. Clin. Pharmacol..

[13] Yuan Q., Wang C., Shi J., Lin Z. (2017). Effects of Ginkgo biloba on dementia: An overview of systematic reviews. J. Ethnopharmacol..

[14] Zhou Y., Li W., Xu L., Chen L. (2011). In Salvia miltiorrhiza, phenolic acids possess protective properties against amyloid beta-induced cytotoxicity, and tanshi-nones act as acetylcholinesterase inhibitors. Environ. Toxicol. Pharmacol..

[15] Wu A., Zeng W., Wong V.K., Zhu Y., Lo A.C.Y., Liu L., Law B.Y. (2017). Hederagenin and α-hederin promote degradation of proteins in neurodegenerative diseases and improve motor deficits in MPTP-mice. Pharmacol. Res..

[16] Wu A., Kam-Wai Wong V., Zeng W., Liu L., Yuen-Kwan Law B. (2015). Identification of novel autophagic Radix Polygalae fraction by cell membrane chromatography and UHPLC-(Q)TOF-MS for degradation of neurodegenerative disease proteins. Sci. Rep..

[17] Wu A., Wong V.K., Xu S., Chan W., Ng C., Liu L., Law B.Y. (2013). Onjisaponin B Derived from Radix Polygalae Enhances Autophagy and Accelerates the Degradation of Mutant α-Synuclein and Huntingtin in PC-12 Cells. Int. J. Mol. Sci..

[18] Wong V.K., Wu A.G., Wang J.R., Liu L., Law B.Y. (2015). Neferine Attenuates the Protein Level and Toxicity of Mutant Huntingtin in PC-12 Cells via Induction of Autophagy. Molecules.

[19] Wang X., Wu J., Yu C., Tang Y., Liu J., Chen H., Jin B., Mei Q., Cao S., Qin D. (2017). Lychee Seed Saponins Improve Cognitive Function and Prevent Neuronal Injury via Inhibiting Neuronal Apoptosis in a Rat Model of Alzheimer’s Disease. Nutrients.

[20] Wang X., Zhang H., Liu J., Chen R., Tang Y., Chen H., Gu L., Li M., Cao S., Qin D. (2017). Inhibitory Effect of Lychee Seed Saponins on Apoptosis Induced by Aβ25-35 through Regulation of the Apoptotic and NF-κB Pathways in PC12 Cells. Nutrients.

[21] Miketova P., Schram K.H., Whitney J., Li M., Huang R., Kerns E., Valcic S., Timmermann B.N., Rourick R., Klohr S. (2000). Tandem mass spectrometry studies of green tea catechins. Identification of three minor components in the polyphenolic extract of green tea. J. Mass Spectrom..

[22] Porter L.J., Newman R.H., Foo L.Y., Wong H., Hemingway R.W. (1982). Polymeric Proanthocyanidins. I3C Nmr Studies of Procyanidins. J. Chem. Soc. Perkin Trans..

[23] Koerner J.L., Hsu V.L., Lee J., Kennedy J.A. (2009). Determination of proanthocyanidin A2 content in phenolic polymer isolates by reversed-phase high-performance liquid chromatography. J. Chromatogr. A.

[24] Latta C.H., Sudduth T.L., Weekman E.M., Brothers H.M., Abner E.L., Popa G.J., Mendenhall M.D., Gonzalez-Oregon F., Braun K., Wilcock D.M. (2015). Determining the role of IL-4 induced neuroinflammation in microglial activity and amyloid-β using BV2 microglial cells and APP/PS1 transgenic mice. J. Neuroinflamm..

[25] Godyń J., Jończyk J., Panek D., Malawska B. (2016). Therapeutic strategies for Alzheimer’s disease in clinical trials. Pharmacol. Rep..

[26] Sevigny J., Chiao P., Bussière T., Weinreb P.H., Williams L., Maier M., Dunstan R., Salloway S., Chen T., Ling Y. (2016). The antibody aducanumab reduces Aβ plaques in Alzheimer’s disease. Nature.

[27] Alarcón R., Fuenzalida C., Santibáñez M., Bernhardi R. (2005). Expression of Scavenger Receptors in Glial Cells: Comparing the adhesion of astrocytes and microglia from neonatal rats to surface-bound β-amyloid. J. Biol. Chem..

[28] Grathwohl S.A., Kälin R.E., Bolmont T., Prokop S., Winkelmann G., Kaeser S.A., Odenthal J., Radde R., Eldh T., Gandy S. (2009). Formation and maintenance of Alzheimer’s disease β-amyloid plaques in the absence of microglia. Nat. Neurosci..

[29] Jantzen P.T., Connor K.E., DiCarlo G., Wenk G.L., Wallace J.L., Rojiani A.M., Coppola D., Morgan D., Gordon M.N. (2002). Microglial Activation and β-Amyloid Deposit Reduction Caused by a Nitric Oxide-Releasing Nonsteroidal Anti-Inflammatory Drug in Amyloid Precursor Protein Plus Presenilin-1 Transgenic Mice. J. Neurosci..

[30] Lull M.E., Block M.L. (2010). Microglial Activation and Chronic Neurodegeneration. Neurotherapeutics.

[31] Dheen S.T., Kaur C., Ling E.A. (2007). Microglial Activation and its Implications in the Brain Diseases. Curr. Med. Chem..

[32] Condello C., Yuan P., Schain A., Grutzendler J. (2015). Microglia constitute a barrier that prevents neurotoxic protofibrillar Aβ42 hotspots around plaques. Nat. Commun..

[33] Liu S., Lin J., Wang C., Chen H., Yang D. (2009). Antioxidant properties of various solvent extracts from lychee (*Litchi chinenesis* Sonn.) flowers. Food Chem..

[34] Sakurai T., Nishioka H., Fujii H., Nakano N., Kizaki T., Radak Z., Iizawa T., Haga S., Ohno H. (2008). Antioxidative Effects of a New Lychee Fruit-Derived Polyphenol Mixture, Oligonol, Converted into a Low-Molecular Form in Adipocytes. Biosci. Biotechnol. Biochem..

[35] Xu X., Xie H., Wang Y., Wei X. (2010). A-Type Proanthocyanidins from Lychee Seeds and Their Antioxidant and Antiviral Activities. J. Agric. Food Chem..

[36] Qi S., Huang H., Huang J., Wang Q., Wei Q. (2015). Lychee (*Litchi chinensis* Sonn.) seed water extract as potential antioxidant and antiobese natural additive in meat products. Food Control..

[37] Heneka M.T., Golenbock D.T., Latz E. (2015). Innate immunity in Alzheimer’s disease. Nat. Immunol..

[38] Tarasoff-Conway J.M., Carare R.O., Osorio R.S., Glodzik L., Butler T., Fieremans E., Axel L., Rusinek H., Nicholson C., Zlokovic B.V. (2015). Clearance systems in the brain-implications for Alzheimer disease. Nat. Rev. Neurol..

[39] Rajmohan R., Reddy P.H. (2017). Amyloid-Beta and Phosphorylated Tau Accumulations Cause Abnormalities at Synapses of Alzheimer’s disease Neurons. J. Alzheimers Dis..

[40] Ferreira S.T., Lourenco M.V., Oliveira M.M., De Felice F.G. (2015). Soluble amyloid-β oligomers as synaptotoxins leading to cognitive impairment in Alzheimer’s disease. Front. Cell. Neurosci..

[41] Couturier J., Paccalin M., Morel M., Terro F., Milin S., Pontcharraud R., Fauconneau B., Page G. (2011). Prevention of the b-amyloid peptide-induced inflammatory process by inhibition of double-stranded RNA-dependent protein kinase in primary murine mixed co-cultures. J. Neuroinflamm..

[42] Zujovic V., Benavides J., Vige X., Carter C., Taupin V. (2000). Fractalkine Modulates TNF-α Secretion and Neurotoxicity Induced by Microglial Activation. Glia.

[43] Faro M.L.L., Fox B., Whatmore J.L., Winyard P.G., Whiteman M. (2014). Hydrogen sulfide and nitric oxide interactions in inflammation. Nitric Oxide.

[44] Tian M., Deng Y.Y., Hou D.R., Li W., Feng X.L., Yu Z.L. (2015). Association of IL-1, IL-18, and IL-33 gene polymorphisms with late-onset Alzheimer’s disease in a Hunan Han Chinese population. Brain Res..

[45] Jia Y., Wang N., Liu X. (2017). Resveratrol and Amyloid-Beta: Mechanistic Insights. Nutrients.

[46] Ehrnhoefer D.E., Bieschke J., Boeddrich A., Herbst M., Masino L., Lurz R., Engemann S., Pastore A., Wanker E.E. (2008). EGCG redirects amyloidogenic polypeptides into unstructured, off-pathway oligomers. Nat. Struct. Mol. Biol..

[47] Yang F., Lim G.P., Begum A.N., Ubeda O.J., Simmons M.R., Ambegaokar S.S., Chen P.P., Kayed R., Glabe C.G., Frautschy S.A. (2005). Curcumin Inhibits Formation of Amyloid β Oligomers and Fibrils, Binds Plaques, and Reduces Amyloid In Vivo. J. Biol. Chem..

[48] Kook Y. (2008). The inhibitory effect of Scutellaria baicalensis on type 1 interferon production in Raw 264.7 cells. Herb. Formula Sci..

[49] Pervin M., Unno K., Ohishi T., Tanabe H., Miyoshi N., Nakamura Y. (2018). Beneficial Effects of Green Tea Catechins on Neurodegenerative Diseases. Molecules.

[50] Toda T., Sunagawa T., Kanda T., Tagashira M., Shirasawa T., Shimizu T. (2011). Apple Procyanidins Suppress Amyloid β-Protein Aggregation. Biochem. Res. Int..

[51] Heo H.J., Lee C.Y. (2005). Epicatechin and Catechin in Cocoa Inhibit Amyloid β Protein Induced Apoptosis. J. Agric. Food Chem..

[52] Laurenzi M.A., Arcuri C., Rossi R., Marconi P., Bocchini V. (2001). Effects of Microenvironment on Morphology and Function of the Microglial Cell Line BV-2. Neurochem. Res..

[53] Liu X., Wu J.Y., Zhou F., Sun X.L., Yao H.H., Yang Y., Ding J.H., Hu G. (2006). The regulation of rotenone-induced inflammatory factor production by ATP-sensitive potassium channel expressed in BV-2 cells. Neurosci. Lett..

[54] Mincheva-Tasheva S., Soler R.M. (2013). NF-κB Signaling Pathways. Neuroscientist.

[55] Alawdi S.H., El-Denshary E.S., Safar M.M., Eidi H., David M., Abdel-Wahhab M.A. (2017). Neuroprotective Effect of Nanodiamond in Alzheimer’s Disease Rat Model: A Pivotal Role for Modulating NF-κB and STAT3 Signaling. Mol. Neurobiol..

[56] Zhong Z., Umemura A., Sanchez-Lopez E., Liang S., Shalapour S., Wong J., He F., Boassa D., Perkins G., Ali S.R. (2016). NF-κB Restricts Inflammasome Activation via Elimination of Damaged Mitochondria. Cell.

[57] Liu B., Sun L., Liu Q., Gong C., Yao Y., Lv X., Lin L., Yao H., Su F., Li D. (2015). A Cytoplasmic NF-κB Interacting Long Noncoding RNA Blocks IκB Phosphorylation and Suppresses Breast Cancer Metastasis. Cancer Cell.

[58] Seo E., Fischer N., Efferth T. (2018). Phytochemicals as inhibitors of NF-κB for treatment of Alzheimer’s disease. Pharmacol. Res..

[59] Park S., Kim M., Kim Y.J., Lee Y., Bae D., Kim S., Na Y., Yoon H. (2015). Selective PCAF inhibitor ameliorates cognitive and behavioral deficits by suppressing NF-κB-mediated neuroinflammation induced by Aβ in a model of Alzheimer’s disease. Int. J. Mol. Med..

[60] Shi Z., Han Y., Han X., Zhang K., Chang Y., Hu Z., Qi H., Ting C., Zhen Z., Hong W. (2016). Upstream regulators and downstream effectors of NF-κB in Alzheimer’s disease. J. Neurol. Sci..

[61] Lian H., Yang L., Cole A., Sun L., Chiang A.C.A., Fowler S.W., Shim D.J., Rodriguez-Rivera J., Taglialatela G., Jankowsky J.L. (2015). NF-κB-Activated Astroglial Release of Complement C3 Compromises Neuronal Morphology and Function Associated with Alzheimer’s Disease. Neuron.

[62] Martire S., Mosca L., d’Erme M. (2015). PARP-1 involvement in neurodegeneration: A focus on Alzheimer’s and Parkinson’s diseases. Mech. Ageing Dev..

[63] Zeng J., Libien J., Shaik F., Wolk J., Hernández A.I. (2016). Nucleolar PARP-1 Expression Is Decreased in Alzheimer’s Disease: Consequences for Epigenetic Regulation of rDNA and Cognition. Neural Plast..

[64] Figueira I., Garcia G., Pimpão R.C., Terrasso A.P., Costa I., Almeida A.F., Tavares L., Pais T.F., Pinto P., Ventura M.R. (2017). Polyphenols journey through blood-brain barrier towards neuronal protection. Sci. Rep..

[65] Schaffer S., Halliwell B. (2012). Do polyphenols enter the brain and does it matter? Some theoretical and practical considerations. Genes Nutr..

[66] Wu L., Zhang Q.L., Zhang X.Y., Lv C., Li J., Yuan Y., Yin F.X. (2012). Pharmacokinetics and Blood-Brain Barrier Penetration of (+)-Catechin and (−)-Epicatechin in Rats by Microdialysis Sampling Coupled to High-Performance Liquid Chromatography with Chemiluminescence Detection. J. Agric. Food Chem..

[67] Neves A.R., Queiroz J.F., Reis S. (2016). Brain-targeted delivery of resveratrol using solid lipid nanoparticles functionalized with apolipoprotein E. J. Nanobiotechnol..

[68] Jain A., Rani V. (2018). Mode of treatment governs curcumin response on doxorubicin-induced toxicity in cardiomyoblasts. Mol. Cell. Biochem..

