# Supplementary material for: Polyphenols Derived from Lychee Seed Suppress Aβ (1-42)-Induced Neuroinflammation
Source: Int J Mol Sci. 2018 Jul 20;19(7):2109. doi: 10.3390/ijms19072109 (PMC6073121; doi:10.3390/ijms19072109)
Supplement: Supplementary file 1 [file ijms-19-02109-s001.pdf]

Supplementary Information

Polyphenols derived from Lychee seed suppress Aβ(1-42)-induced neuroinflammation

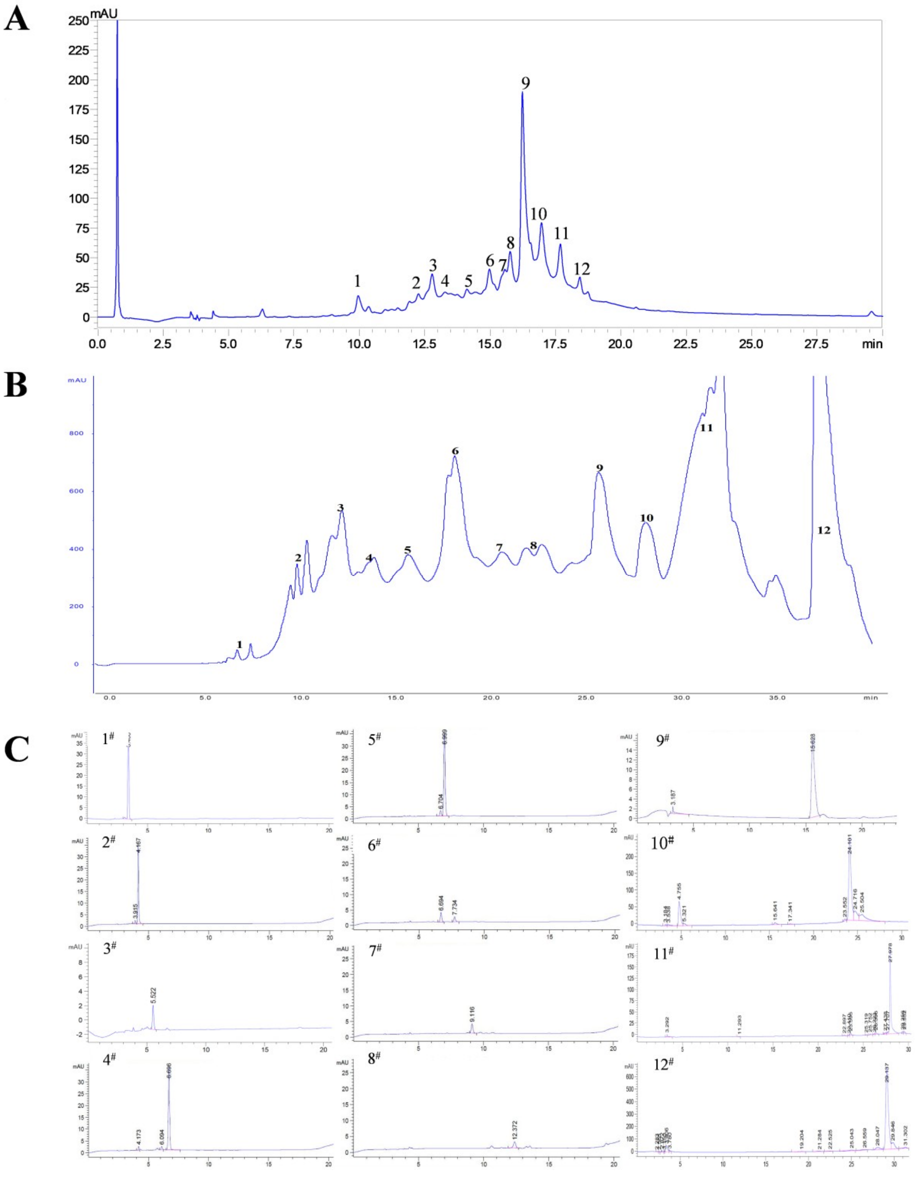

Figure S1. The isolation and purification of the bioactive components in LSF. (A) The chemical fingerprint of LSF was analyzed at 280 nm by a Shimadzu HPLC system which comprised of a DGU-20A5R degasser, two LC-20ADXR pumps, a SIL-20ACXR autosampler, a CTO-20AC column oven, a SPD-M20A diode array detector and a Shimadzu CBM-20A system controller. Chromatographic analyses were achieved at 35 °C with a C18 column (GL science, 5.0 μm particle size, 4.6 mm×250 mm), using acetonitrile and water-acetic acid (99.9:0.1, v/v) as the mobile phase A and phase B, respectively. The delivered rate of mobile phase was 1.0 mL/min with the injection volume of 20 μL. For the gradient separation, the process was set as follows: 0-10 min, 5%-24% (A), 10-11 min, 24%-24% (A), 11-11.01 min, 24%-28% (A), 11.01-12 min, 28%-28% (A), 12-12.01 min, 28%-36% (A),

12.01-13 min, 36%-36% (A), 13-13.01 min, 36%-40% (A), 13.01-14 min, 40%-40% (A), 14-14.01 min, 40%-45% (A), 14.01-15 min, 55%-55% (A), 15-15.01 min, 55%-50% (A), 15.01-16 min, 50%-50% (A), 16-18 min, 50%-60% (A), 18-30 min, 60%-70% (A). The data were analyzed with LabSolutions software. (B) The separation chromatogram of the components in LSF were obtained by the ÄKTA purifier protein purification system and collected 12 fractions, (C) The chromatograms of the 12 isolated fractions were obtained by HPLC chromatography.

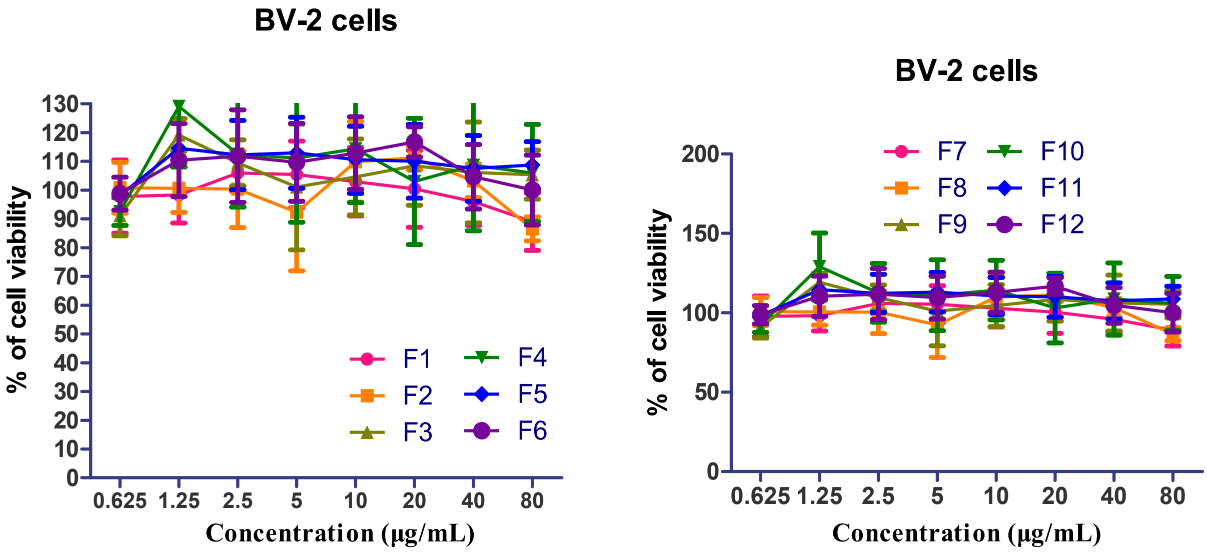

Figure S2. Cytotoxicity of 12 isolated fractions from LSF against BV-2 cells for 48 h by using CCK-8 kit. In brief, BV-2 cells were plated on 96-well plates (100 μL, 1×10^5 cells/well) one day before LSF treatment under the indicated concentrations. After 48 h treatment, 10 μL of CCK-8 solution was added into each well and incubated for another 2 h. Colorimetric reading of the solute mixture was then determined at 450 nm using a standard plate-reader (DG5032, Hua Dong, Nanjing, China). The percentage of cell viability was calculated using the following formula: Cell viability (%) = Cells number $_{\text{treated}}$/Cells number $_{\text{DMSO control}}$ × 100. Data were obtained from three independent experiments.

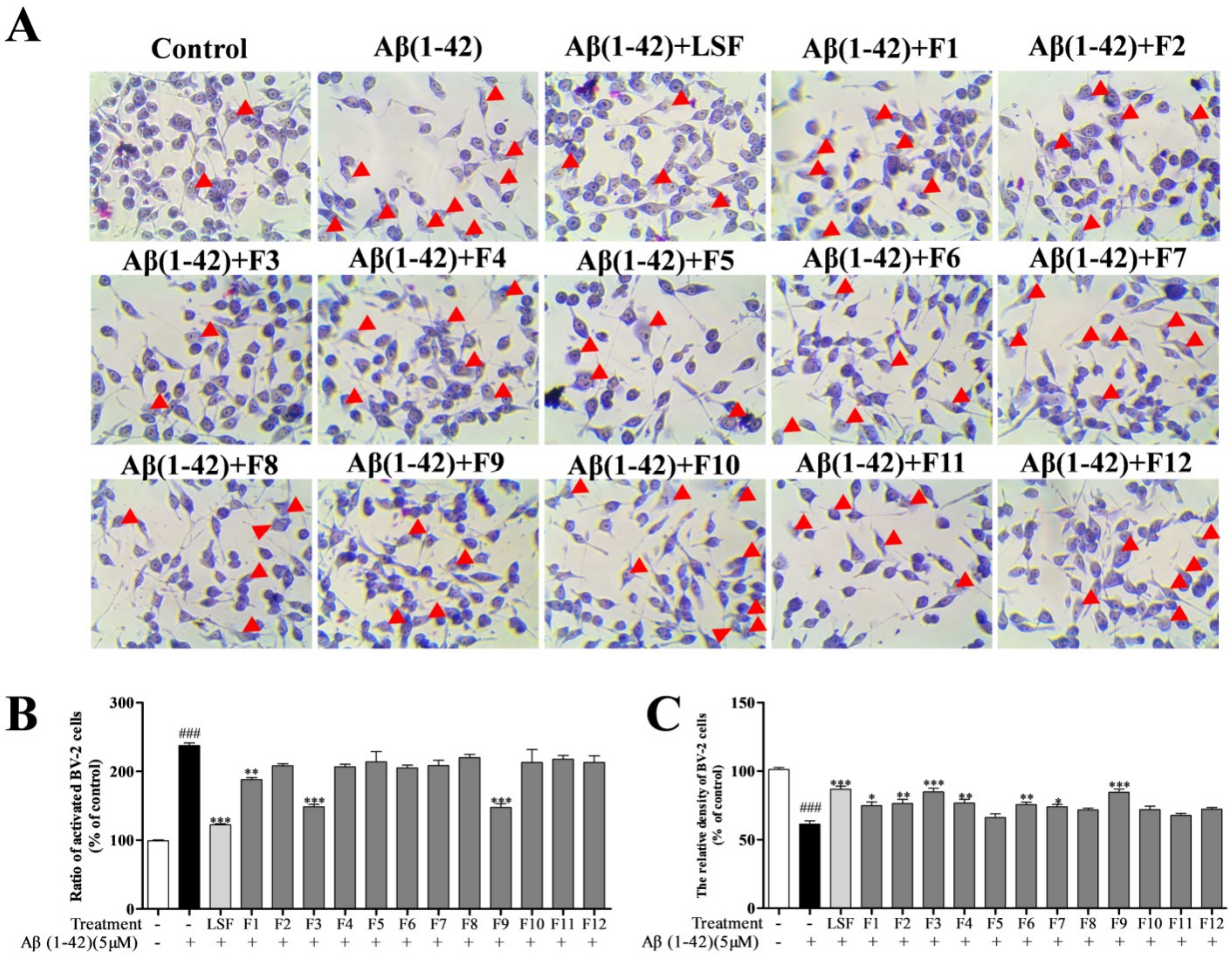

Figure S3. The morphology of BV-2 cells treated with LSF or LSF derived 12 isolated fractions. BV-2 cells were pretreated with 5 μM Aβ(1-42) for 12 h, then followed with incubations of LSF (0.469 μg/mL) and fraction 1-12 (μg/mL) for another 12 h. (A) The red arrows indicate the activated BV-2 cells with ameboid shape. Magnification: ×100; Scale bar: 50 μm. (B) The ratio of activated BV-2 cells. It was calculated by counting the number of the activated BV-2 cells in all of BV-2 cells views. (C) The density of BV-2 cells. It was quantified by counting the number of all of BV-2 cells. A minimum of 150 cells from 3 randomly selected fields were scored. The BV-2 cells treated with medium alone were set as the control group, and the BV-2 cells treated with 5 μM Aβ(1-42) alone were set as the Aβ group. ### $P < 0.001$ vs. Control group; * $P < 0.05$, ** $P < 0.01$, *** $P < 0.001$ *vs.* Aβ group.

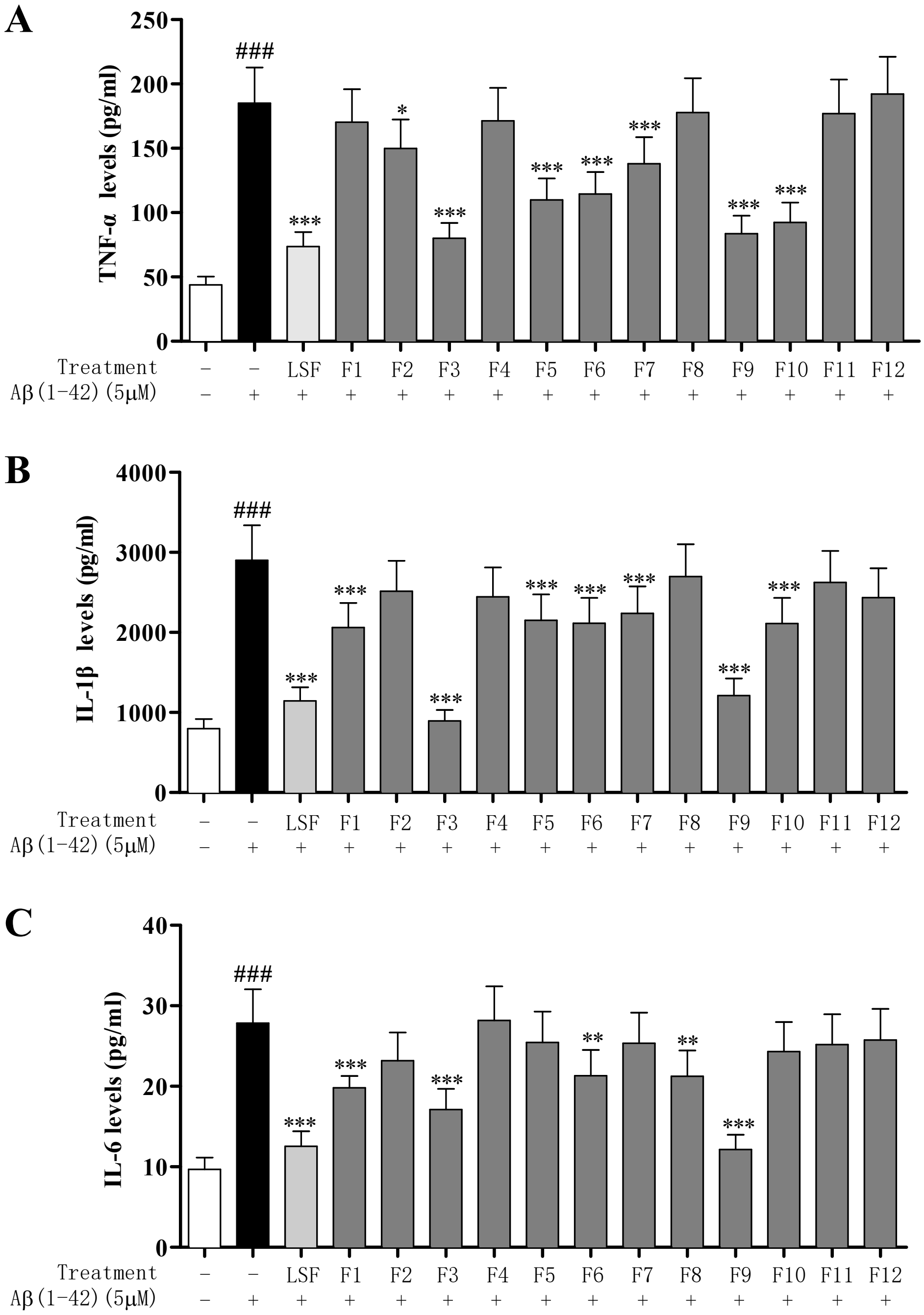

Figure S4. The secretion of TNF-α, IL-1β and IL-6 in activated BV-2 cells treated with or without LSF and 12 isolated fractions. BV-2 cells were pretreated with 5 μM Aβ(1-42) for 12 h, then followed with incubations of LSF (0.469 μg/mL) and fraction 1-12 (μg/mL) for another 12 h. TNF-α (A), IL-1β (B) and IL-6 (C) levels in supernatants were determined by ELISA as described in Materials and Methods. The BV-2 cells treated with medium alone were set as the control group, and the BV-2 cells treated with 5 μM Aβ(1-42) alone were set as the Aβ group. These results were expressed as mean ± SD. ### $P < 0.001$ *vs.* control; * $P < 0.05$, ** $P < 0.01$, *** $P < 0.001$ *vs.* Aβ group.

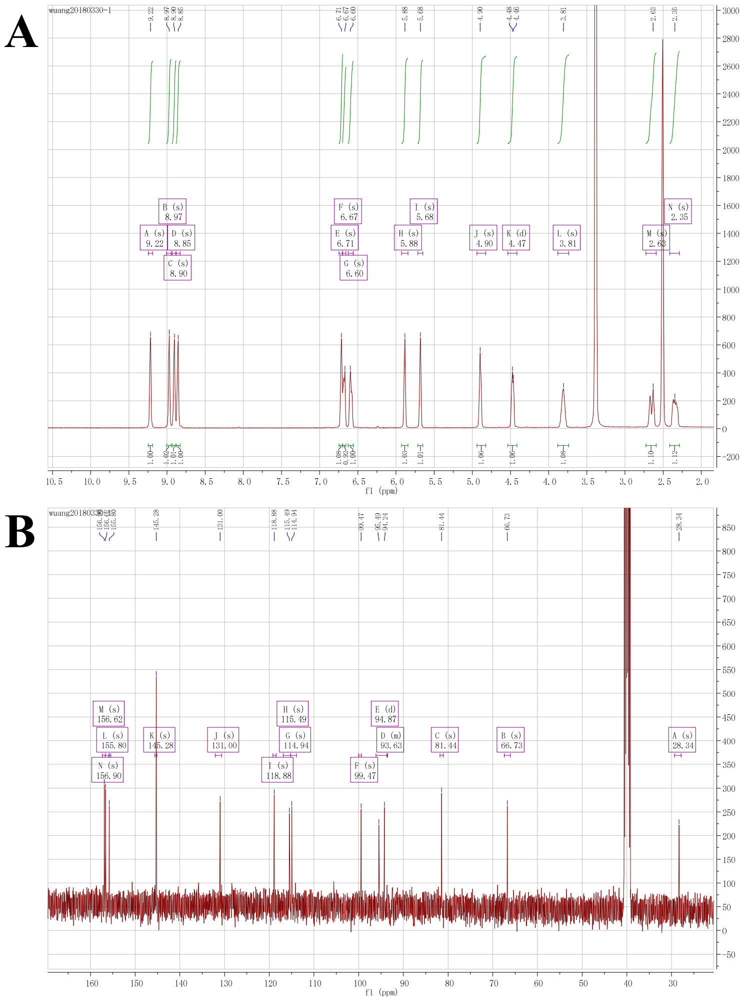

Figure S5. ^{1}H NMR and ^{13}C NMR spectrums of the compound isolated from fraction 3. (**A**) ^{1}H NMR spectrum of catechin, (**B**) ^{13}C NMR spectrum of catechin.

A

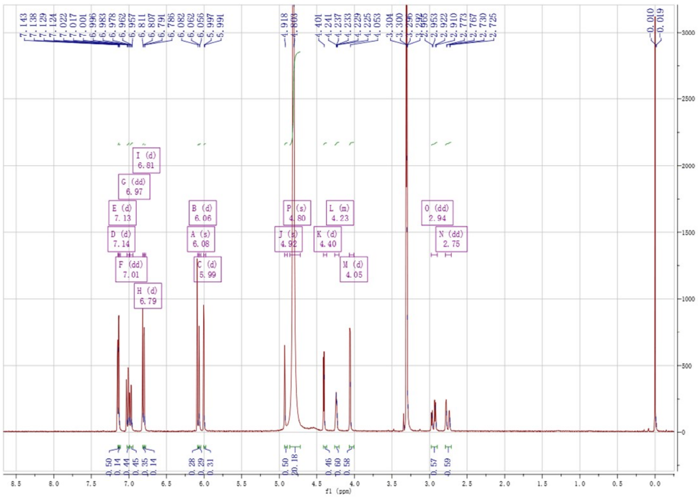

B

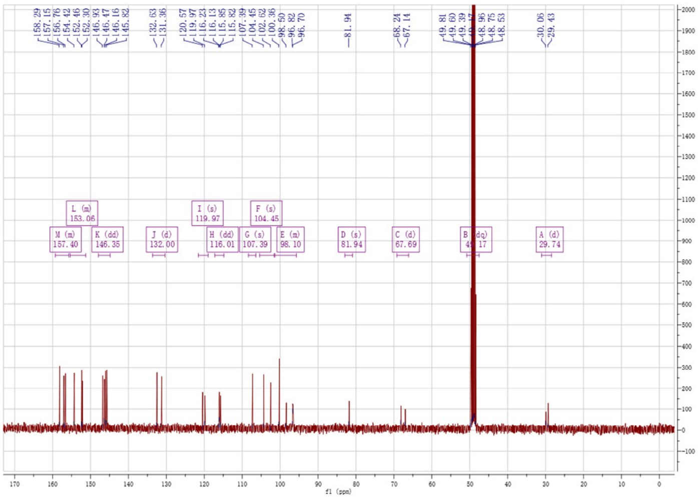

Figure S6. ^{1}H NMR and ^{13}C NMR spectrums of the compound isolated from fraction 9. (**A**) ^{1}H NMR spectrum of procyanidin A2, (**B**) ^{13}C NMR spectrum of procyanidin A2.

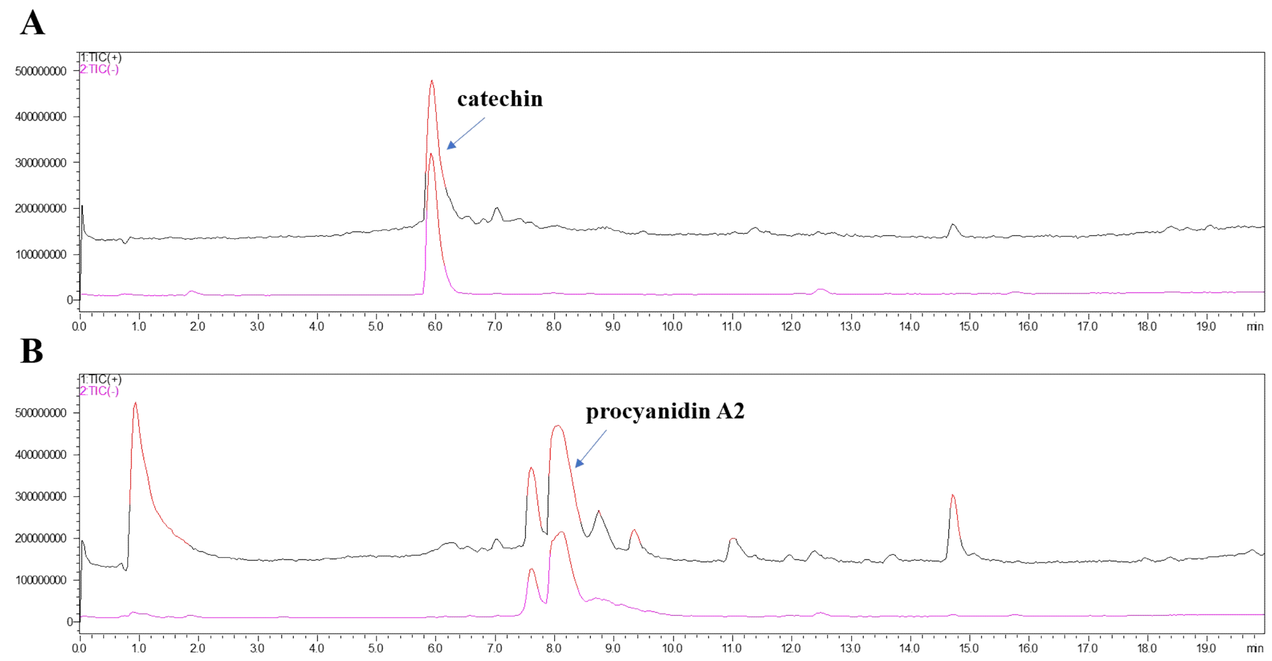

Figure S7. The total ion chromatograms (TIC) of (A) catechin and (B) procyanidin A2 by using LC-ESI-MS/MS in positive and negative mode.

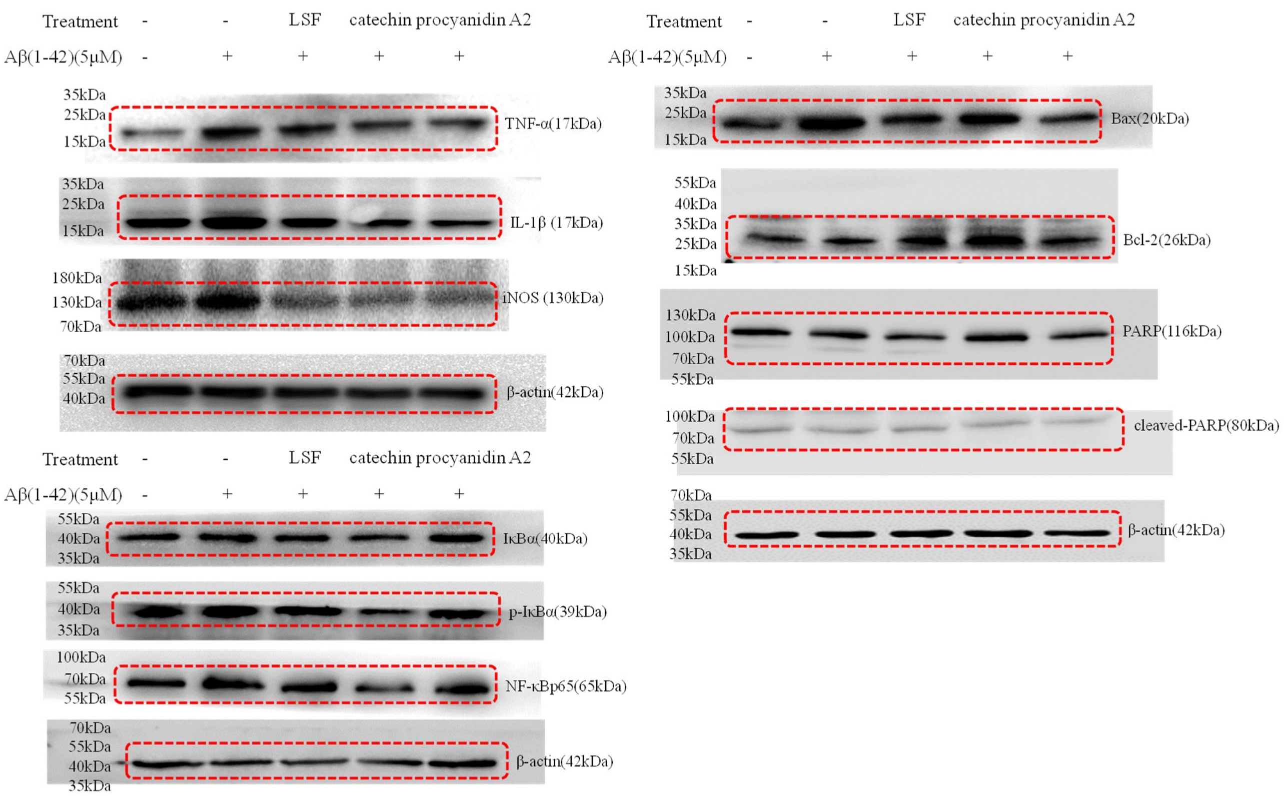

Figure S8. The full-length Western blotting images.
